# *ERV14* receptor impacts mycelial growth *via* its interactions with cell wall synthase and transporters in *Aspergillus niger*

**DOI:** 10.3389/fmicb.2023.1128462

**Published:** 2023-04-11

**Authors:** Junwei Zheng, Linlin Yao, Xu Zeng, Bin Wang, Li Pan

**Affiliations:** ^1^School of Biology and Biological Engineering, South China University of Technology, Guangzhou, China; ^2^Guangdong Provincial Key Laboratory of Fermentation and Enzyme Engineering, South China University of Technology, Guangzhou, China

**Keywords:** *Aspergillus niger*, secretory pathway, *Erv14*, high-throughput Y2H screening, transporters, quantitative proteome

## Abstract

Efficient protein secretion is closely correlated with vesicle sorting and packaging, especially with cargo receptor-mediated selective transport for ER exit. Even though *Aspergillus niger* is considered an industrially natural host for protein production due to its exceptional secretion capacity, the trafficking mechanism in the early secretory pathway remains a black box for us to explore. Here, we identified and characterized all putative ER cargo receptors of the three families in *A. niger.* We successfully constructed overexpression and deletion strains of each receptor and compared the colony morphology and protein secretion status of each strain. Among them, the deletion of *Erv14* severely inhibited mycelial growth and secretion of extracellular proteins such as glucoamylase. To gain a comprehensive understanding of the proteins associated with *Erv14*, we developed a high-throughput method by combining yeast two-hybrid (Y2H) with next-generation sequencing (NGS) technology. We found *Erv14* specifically interacted with transporters. Following further validation of the quantitative membrane proteome, we determined that *Erv14* was associated with the transport of proteins involved in processes such as cell wall synthesis, lipid metabolism, and organic substrate metabolism.

## Introduction

The secretory pathway of fungi is responsible for delivering large amounts of newly synthesized proteins to their proper cellular location and is essential for cellular function. First, nascent peptides are translocated into the endoplasmic reticulum in a co-translational manner or post-translational manner. In the ER, proteins undergo folding and glycosylation and then are packaged into the Golgi apparatus for further processing until being mature. Next, they are transported to various compartments, including the endosome, vacuole, plasma membrane, etc. The trafficking between different parts generally depends on vesicles, and the transport from the endoplasmic reticulum (ER) to the Golgi apparatus is mediated by Coat Protein Complex II (COPII) (Kurokawa and Nakano, 2019). The COPII budding machinery is relatively conserved in eukaryotes. Initially, the guanine nucleotide exchange factor Sec12p activates the cytoplasmic GTPase Sar1p, causing it to insert the N-terminal amphiphilic α-helix into the endoplasmic reticulum membrane (Barlowe and Schekman, 1993). Next, Sar1p-GTP recruits the Sec23p-Sec24p complex as the inner layer, and then the Sec13p-Sec31p complex is wrapped in its outer layer (Stagg et al., 2006; Bhattacharya et al., 2012). In addition to structural functions, Sec24p also acts as a protein cargo adaptor, which can not only directly bind transmembrane proteins but also recruit protein sorting receptors to bind soluble proteins (Pagant et al., 2015). Sec23p, the Sar1-specific GTPase activating protein (GAP), can hydrolyze the GTP on Sar1p to trigger vesicles to detach from the ER membrane (Bonifacino and Glick, 2004).

Protein sorting receptors of the vesicles are crucial for such a large amount of protein to be transported out of the ER in an orderly manner (Barlowe and Helenius, 2016). Protein sorting receptors are mainly divided into three families: ERGIC53, p24, and ERV family. Each family of receptors has been reported to be associated with the transport of specific cargo proteins. The ERGIC53 family members Emp47p and Vip36p are considered to be the specific receptors for glycoproteins (Kamiya et al., 2008). The receptors of the p24 family can interact with each other, and they have been reported to transport GPI proteins (Tashima et al., 2022). Different from the first two receptor family members, the ERV family members are multi-transmembrane proteins, and the type of protein transported by each receptor is not consistent (Dancourt and Barlowe, 2010). Erv29p is associated with the ER exit of glycosylated alpha-factor pheromone precursor (gpαf) and carboxypeptidase Y (CPY) (Belden and Barlowe, 2001). Svp26p facilitates the transport of a set of mannosyltransferases in yeast (Noda and Yoda, 2010). Erv14p assists in the transport of multiple transmembrane proteins involved in polarized growth (Powers and Barlowe, 2002).

*Aspergillus niger* is considered an industrially natural host for protein production due to its exceptional secretion capacity (Cairns et al., 2018), but little is known about its receptors. In this study, we first identified all receptors of each family in *A. niger* by homology alignment and phylogenetic analysis. We successfully constructed each receptor knockout strain and overexpression strain and analyzed the effect of each receptor on phenotype and extracellular protein secretion. Among them, the deletion of *Erv14* severely inhibited mycelial growth and secretion of extracellular proteins such as glucoamylase. The interactome of *Erv14* identified by high-throughput Y2H screening revealed that it interacts with a variety of transmembrane transporter proteins. The membrane proteome also provided further evidence that multiple proteins associated with the cell wall and lipid metabolism were reduced due to the deletion of *Erv14*. Our data provided a basis for future studies of the mechanisms underlying receptor-mediated vesicle trafficking in the early secretory pathway.

## Materials and methods

### Strains and cultural conditions

*Escherichia coli* strain Mach1 T1 was used for plasmid construction and cultivated in Luria–Bertani (LB) broth. *A. niger* CBS513.88 (△kusA△pyrG) was used as the host strain for gene manipulation (Dong et al., 2021). All *Aspergillus* strains were generally cultivated on Czapek-Dox (CD) medium (Ozeki et al., 1994). High osmotic pressure Czapek–Dox (HCD) medium with sucrose (1 M) was used for protoplast transformation. Dextrose peptone yeast extract (DPY) liquid medium was used for DNA extraction and RNA isolation (Imanaka et al., 2010). Complete medium (CM) was used for the observation of mycelial growth, which consisted of starch (1% w/v), peptone (1% w/v), beef extract (0.3% w/v), yeast extract (0.2% w/v), and sodium chloride (0.2% w/v). For fermentation, all *Aspergillus* strains were cultivated in the shake flask medium as described previously reported (Lin et al., 2020). *Saccharomyces cerevisiae* strain NMY51 was used as the parental strain for the Y2H screening. SD medium with different DO supplements used for the screening and cultivation of hybrid yeast strains.

### Plasmid construction

We pre-constructed a universal expression vector based on the pMD18T plasmid. The promoter and terminator are Ptef and Ttef, left with the enzyme cut site BglII in the middle, followed by ANpyrG as the screening marker, and the upstream and downstream 1,000 bp fragment of the *pyrG* as the homology arm at both ends. All DNA fragments were amplified with the high-fidelity polymerase PrimeSTAR (Takara, Japan) following the manual standardized procedure, and ligated to the pMD18T plasmid using the NEBuilder HiFi DNA Assembly Cloning Kit (NEB, Ipswich, United States). To construct receptor overexpression plasmids, the gene for each receptor was amplified from the genome and inserted into the universal vector linearized by BglII. To construct fluorescently labeled mstE and snf3 plasmids, mstE and snf3 cDNAs and GFP were fused and inserted into the middle of Ptef-Ttef. The pyrithiamine resistance gene ptrA followed the expression cassette and was flanked by a 1,500 bp homology arm at the outermost edge for integration into the *A. niger* genomic AM270982:171718-174718 locus. To construct the Y2H bait plasmid, the cDNA of *Erv14* was amplified by PCR, and the PCR product was constructed into the linearized plasmid pBT-STE, which was digested with SfiI. The prey plasmid library was constructed by Shanghai Biogene Biotech company. Here, the whole cDNA was integrated with the linearized plasmid pPR3-NubG, which was digested with SfiI.

### Knockout of each receptor *via* CRISPR/Cas9 technology

The sgRNAs for target genes were searched by the online tool Cas-Designer (CRISPR RGEN Tools)^1^ and screened for base preference at each position of the 20 nt protospacer sequence (Doench et al., 2014). All sgRNA sequences targeting each receptor were listed in Supplementary Table S1. The ribonucleoprotein (RNP)-based CRISPR-Cas9 method was used for the genetic manipulation of *A. niger* as recently reported (Yang et al., 2020). The RNP complex was pre-assembled from 6 μg of SpCas protein and 10 μg of *in vitro* synthesized sgRNA, which was subsequently transformed into protoplasts along with 100ug of donor DNA fragments. The donor DNA was inserted into the genome in the microhomology-mediated end joining (MMEJ) manner (Zhang et al., 2016), which consisted of the middle selection marker *ANpyrG* and a 39 bp homologous arm at both ends.

### Microscopy

To observe spore germination, 10^4^ spores were inoculated into 500 μl of CD liquid medium on coverslips and incubated for 40 h at 30°C. Carefully removed the medium, and gently washed with PBS buffer. 50 μl of PBS buffer was added to the glass slide and the coverslip with mycelium was pressed over it. To observe the localization of monosaccharide transporters in the control strain and the DEL-ERV14 strain, 10^4^ spores were inoculated into 500 μl of DPY liquid medium on coverslips and incubated for 12 h at 30°C. To observe conidiophore, 10^4^ spores were inoculated around a small block of CD medium on a slide, and then covered with a coverslip and incubated on a plate for 2 days. All images were observed with a Zeiss Axio Imager A2 microscope equipped with a 100× oil objective lens.

### Secretion assays

All *Aspergillus* strains were cultivated in the shake flask medium at 30°C for 6 days. 2 ml sample was taken and centrifuged at 12,000 g at 4°C for 5 min, and the supernatant was used for subsequent experiments. The total protein in the culture supernatant was determined *via* BCA Protein Assay Kit (GBCBIO, Guangzhou, China). Glucoamylase enzymatic activity was measured as described previously reported (Zhu et al., 2017). The dry weight was measured as previously reported (Fiedler et al., 2018). Each experiment was performed with two biological replicates. Statistical analysis was performed by one-way ANOVA. *p < 0.05, **p < 0.01, ***p < 0.001, ****p < 0.0001, *p* values were obtained using Tukey’s multiple comparison test.

### Yeast two-hybrid assay

We chose the mating-based split-ubiquitin system (mbSUS) alternative to the classical yeast two-hybrid (Y2H) system in particular (Grefen et al., 2009). The workflow of the high-throughput Y2H screening is the following: RNA was extracted from *A. niger* cultured in three different media, then cDNA was synthesized and ligated to the prey vector after the addition of an adaptor. The prey library was transformed into the bait strain and then painted in amino acid-deficient media. Ten transformants were picked in one tube within 50 μL ddH_2_O, which was used directly as a template for PCR. Then the target genes were amplified. Finally, all the PCR products were mixed for NGS sequencing.

Y2H Data analysis was divided into three parts: quality control reads matching back to the reference genome, and gene expression quantification. (1) Quality control: FastQC (v0.11.9) was used to check whether the junctions of the reads obtained from sequencing were removed cleanly; (2) matching: Hisat2 (version 2.2.1) was used to match the reads back to the reference genome to obtain sam files, and Samtools was used to convert the sam files into bam files and filter out the unmatched and low-quality reads. Use Samtools to convert the sam files into bam files and filter out the unmatched and low-quality reads with the following parameters: view -F4 -q 30 -bS, and use the sort function in Samtools to sort the bam files; (3) the number of reads on the match is set with the parameters: -T 10 -p -t gene -g gene_id.

### Membrane proteome

Three biological replicates of DEL-Erv14 and control strains were incubated in shake flasks with fermentation medium for 5 days. Mycelium was collected by vacuum pump, washed twice with 0.8 M NaCl and water, and put at −80°C for lyophilization. The lyophilized blocks were ground into the powder with liquid nitrogen and then placed in the tissue grinding tube with cell lysis solution and PMSF for further extraction. The lysate was transferred to a centrifuge tube and centrifuged at 12,000 g for 10 min at 4°C. The supernatant was considered as soluble protein, while the sediment was used to extract membrane proteins. The subsequent steps followed the protocol of the filamentous fungal membrane protein extraction kit (HR9086, Biorab, Beijing, China). The extracted membrane proteins were sent to GeneCreate company (Wuhan, China) for mass spectrometry detection and analysis.

## Results

### Characterization of putative ER cargo receptors in *Aspergillus niger*

**Figure 1:**
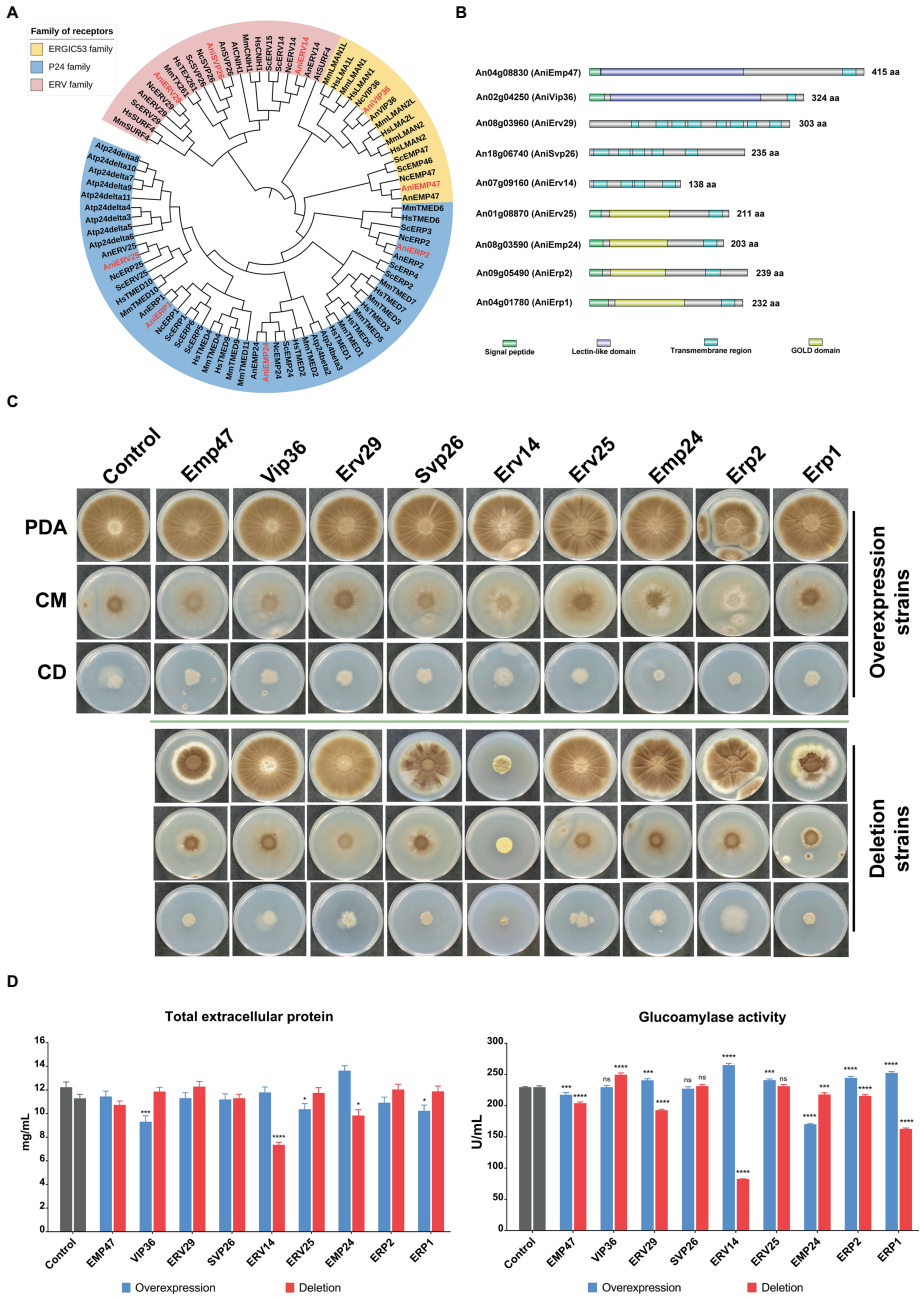
Characterization of putative cargo receptors of different families in *Aspergillus niger.* **(A)** Phylogenetic analysis of different receptor proteins in typical species and comparison of conserved regions of different receptors in *A. niger*. All protein sequences were obtained from the Uniprot database and FIGURE 1 (Continued)aligned by the Clustal Omega. The phylogenetic tree was generated using the neighbor-joining method in MEGA 6 and exported into the Interactive Tree of Life [iTOL: Interactive Tree of Life (embl.de)] for further annotation. The different families were represented by the three background colors of pink, yellow and blue respectively, while the font color of the receptor proteins of *A. niger* was red. Hs, *Homo sapiens*; Mm, *Mus musculus*; At, *Arabidopsis thaliana*; Sc, *Saccharomyces cerevisiae*; Nc, *Neurospora crassa*; An, *Aspergillus nidulans*; Ani, *Aspergillus niger*. The signal peptide and transmembrane regions were predicted by SignalP-6.0 (SignalP - 6.0 - Services - DTU Health Tech) and DeepTMHMM (DTU/DeepTMHMM – BioLib), respectively. The lectin-like domain of the ERGIC53 family and the GOLD domain of the p24 family were predicted by searching for conserved domains in the NCBI database. **(B)** Growth phenotypes of the different receptor mutant strains. 10^4^ spores of various mutants were spotted on plates with different mediums, and mycelial morphology was observed after 7 days of culture at 30°C. CBS Δku strain was used as the control. To detect the effects of cargo receptors deletion or overexpression on extracellular protein secretion in *A. niger*, various mutants and the control strain were cultivated in the shake flask at 30°C for 6 days. The supernatant was collected after centrifuging the culture broth, and total proteins **(C)** and glucoamylase activities **(D)** in the supernatant were quantified *via* BCA Protein Assay Kit and pNPG method, respectively.

To determine ER cargo receptors of *A. niger*, we searched for ER receptor homologous genes in the genomes of *Homo sapiens*, *Mus musculus*, *Arabidopsis thaliana*, *Saccharomyces cerevisiae*, *Neurospora crassa*, *Aspergillus nidulans*, and *A. niger*. Nine putative receptors in *A. niger* were identified. Phylogenetic analysis revealed that these receptors were mainly divided into three clusters representing three families (Figure 1A). *Emp47* (*An04g08830*) and *Vip36* (*An02g04250*) belonged to the ERGIC53 family; while *Erv29* (*An08g03960*), *Svp26* (*An18g06740*), and *Erv14* (*An07g09160*) belonged to the ERV family. The p24 family consisted of numerous members which are divided into four main subfamilies - α, β, γ, and σ. Except for that of *Arabidopsis thaliana*, each p24 protein in each subfamily showed relatively good homology in all species. Only *Erv25* (*An01g08870*), *Emp24* (*An08g03590*), *Erp2* (*An09g05490*), and *Erp1* (*An04g01780*) belonged to each subfamily, respectively, in *A. niger*, which is much less than those of the mammals and yeast (Supplementary Table S2).

To explore the characteristics of the receptors in each family, we predicted the functional domains of nine receptors in *A. niger*. Both Emp47 and Vip36 of the ERGIC53 family contained a signal peptide, a Lectin-like domain, and a transmembrane region. The p24 family receptors, in addition to the conserved GOLD domain, also contained a signal peptide and a transmembrane region (Figure 1A). The ERV family receptors were characterized by the presence of multiple transmembrane regions, although the length and position of each transmembrane region were variable. Furthermore, we used deep learning tools AlphaFold2 to predict the 3D structural models of individual receptors. Each receptor, especially its conserved regions, was well-matched with those of its homologous proteins in *S. cerevisiae* (Supplementary Figure S1).

Overall, *A. niger* possessed three major families of receptors similar to those of other species such as *S. cerevisiae*, but differences in receptor structure and number might indicate differences in function.

### Effects of putative receptors on the colony morphology and protein secretion of *Aspergillus niger*

To investigate the function of each putative receptor in *A. niger*, we constructed knockout and overexpression strains of each receptor. These strains and the control strain were inoculated on the plate with the sporulating medium (PDA), minimal medium (CD), and completed medium (CM) to observe mycelial growth (Figure 1B). Most of the overexpression strains showed no significant phenotypic changes compared to the control strains, except for the OE-Erp2 strain which appeared to grow weakly on the complete medium. However, the deletion of individual receptors from different families resulted in different effects on mycelial growth. In the ERGIC-53 family, the colony size of the DEL-Emp47 mutant was significantly smaller compared to that of the DEL-Vip36 mutant and the control strain on the PDA medium and complete medium, and the mycelium of DEL-Emp47 was not as dispersed as they were on minimal medium. In the ERV family, the DEL-Erv29 mutant showed weak spore production on the PDA medium and complete medium. The colony of the DEL-Svp26 strain showed sectors on the PDA medium, while it did not differ significantly from the control strain on the other media. The DEL-Erv14 mutant exhibited particularly pronounced phenotypic alterations in all three mediums. It had a more aggregated colony morphology and a significantly reduced number of spores. In the p24 family, the DEL-Erp1 mutant showed a marked reduction in growth in both mediums. However, the DEL-Erv25, the DEL-Emp24, and the DEL-Erp2 mutants did not show significant differences, and even the DEL-Erp2 strain grew better on the minimal medium compared to the control strain. To sum up, the deletion of *Vip36, Erv25, and Emp24* had no effect on mycelial growth, and the deletion of *Emp47, Erv29, Svp26*, and *Erp1* slightly slowed down strain growth, while the deletion of *Erp2* promoted strain growth on minimal medium. The growth of the DEL-Erv14 mutant was severely suppressed and was significantly inferior to the other mutant and control strains in terms of colony size and sporulation capacity.

*A. niger* is considered an industrially natural host for protein production due to its exceptional secretion capacity, and how the receptor affects its extracellular protein secretion caused our concern. All the receptor overexpression strains and the deletion mutants as well as the control strain were fermented in shake flasks and sampled on the sixth day. After measuring the total protein in the supernatant of the fermentation broth, we found that overexpression of *Emp47, Erv29, Svp26*, and *Erv14* had almost no impact, while overexpression of *Vip36, Erv25, Erp2*, and *Erp1* showed a 10–20% decrease. The OE-Emp24 strain was the only one with an elevated total extracellular protein yield of 15% compared to the control strain. Among the receptor deletion strains, the DEL-Erv14 strain declined the most significantly, and the DEL-Emp24 decreased by 20%, while the other strains did not show much difference (Figure 1C). Furthermore, glucoamylase activity was also measured as a representative of the endogenous secreted proteins of *A. niger*. Overexpression of individual receptors did not seem to have much effect on promoting the secretion of glucoamylase. The highest elevation was only 15% in the OE-Erv14 strain, while it was even reduced by 30% in the OE-Emp24 strain. However, the glucoamylase activity varied among the deletion mutants, the DEL-Vip36 had higher enzyme activity than the wild strain, while the DEL-Svp26, the DEL-Erv25, the DEL-Emp24, and the DEL-Erp2 were almost unchanged. Knockout of *Emp47, Erv29, Erv14*, and *Erp1* inhibited the secretion of glucoamylase in varying degrees, with the enzyme activity of the DEL-Erv14 strain reduced by about 70% (Figure 1D).

On the whole, overexpression of receptors had limited effects on mycelial morphology and protein secretion, while deletion of some receptors such as *Erv14* and *Erp1* suppress hyphae growth and glucoamylase secretion.

### Deletion of *Erv14* delayed spore germination and protein secretion in *Aspergillus niger*

**Figure 2:**
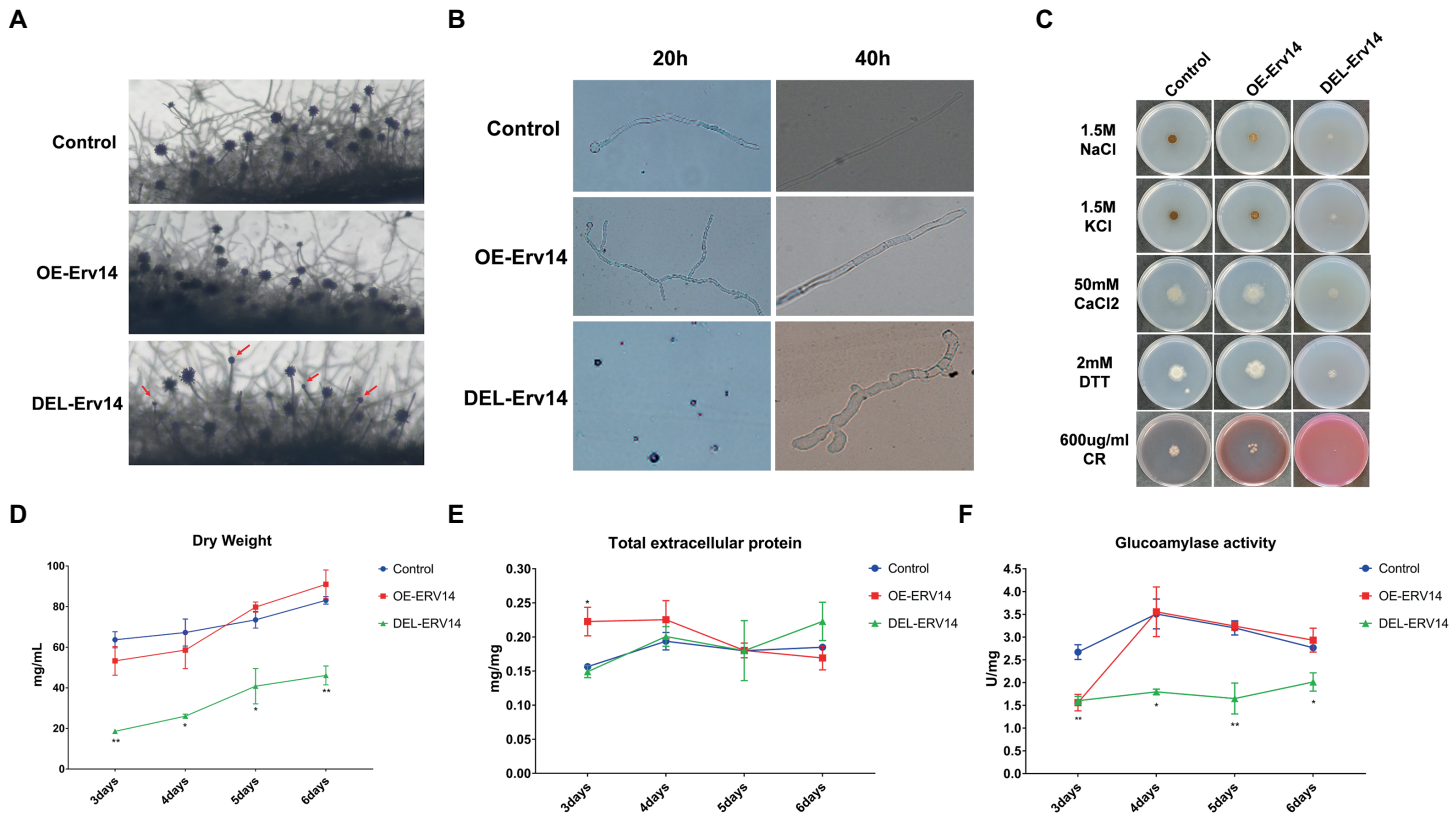
Effect of knockout and overexpression of ERV14 on spore germination and protein secretion in *A. niger*. Comparison of the morphology of the conidial peduncle **(A)** and germination of spores **(B)** of OE-Erv14 and DEL-Erv14 strains. **(C)** Morphological phenotypes of the OE-Erv14, and DEL-Erv14 strains on plates with different environmental stress. The control and mutant strains were cultivated on CD medium with different drugs for 7 days (KCl, NaCl, CaCl_2_ for salt ion pressure, DTT for reducing pressure, and CR(Congo Red)for cell wall interference). Consecutive measurements of dry weight **(D)**, total extracellular protein **(E)** and glucoamylase enzyme activity **(F)** in extracellular proteins of OE-Erv14 and DEL-Erv14 strains from 3 to 6 days. CBS Δku strain was used as the control. The values of secreted protein and enzyme activity were normalized by dry weight.

Since *Erv14* appeared to be the most significant influence from the above characterization of the entire ER receptor, we decided to reveal its function in mycelial growth and protein secretion in detail. First, we observed the conidial peduncles of the OE-Erv14 and the DEL-Erv14 strains on the solid medium. Compared with the control strain, the OE-Erv14 strain showed no significant difference, while the DEL-Erv14 strain showed a reduced number of conidial peduncles and some conidial heads were defective or even naked (Figure 2A). In the liquid medium, spore germination of DEL-Erv14 strain was also significantly delayed compared to the control strain and overexpression strain. After 20 h of inoculation, the spores of the control strain had germinated into mycelium, and the spores of the OE-Erv14 strain had germinated into mycelium with multiple branches, while the DEL-Erv14 strain was still spore-like. After 40 h of incubation, different from the germling tube of the control and the overexpression strains that maintained polarized extension, the spores of the deletion strain sprouted into short but expanded mycelium (Figure 2B). Furthermore, we tested the tolerance of the overexpression and the deletion strains under the high salt, reduction, and cell wall stresses. The mycelium of all strains aggregated in the presence of high Na^+^ and K^+^ salt ion concentrations, although the DEL-Erv14 strain showed more significant changes than the control strain. In the medium with 50 mM CaCl_2_ or 2 mM DTT, the OE-Erv14 strain grew better than the control strain, while the DEL-Erv14 strain grew weaker. Under cell wall stress, the Congo Red completely inhibited the growth of the DEL-Erv14 mutant and had a few effects on the OE-Erv14 strain (Figure 2C). On the other hand, we speculated the growth of the OE-Erv14 and the DEL-Erv14 strains in liquid fermentation by continuous measuring changes in dry weight, total extracellular protein and glucoamylase. The biomass of DEL-Erv14 strain was significantly less than the other two strains during the whole fermentation process. The growth trend of OE-Erv14 strain was similar to that of the control strain, except that the growth was slower in the early stage (Figure 2D). To prevent interference from differences in biomass, we normalized total secreted protein and glucoamylase enzyme activity by dry weight and then compared them. In terms of total proteinper unit dry weight, the amount of the OE-Erv14 strain was highest in the early growth phase and then decreased. In contrast, the DEL-Erv14 strain instead had a relatively high amount of protein on the sixth day (Figure 2E). In terms of glucoamylase, the enzyme activity of both the control and the OE-Erv14 strain reached a peak on the fourth day and then decreased. The enzyme activity of strain DEL-Erv14 maintained at a low level but elevated a little on the sixth day (Figure 2F). In a word, the DEL-Erv14 strain showed significant phenotypic changes - delayed germination, deformed mycelium, poor tolerance to environmental stress. Moreover, although the protein secretion level in the DEL-Erv14 strain was decreased, it might be due to the slow growth of the mycelium, rather than directly affecting the transport of secreted proteins.

### Establishment of a high-throughput Y2H screening method and identification of Erv14-interacting proteins

**Figure 3:**
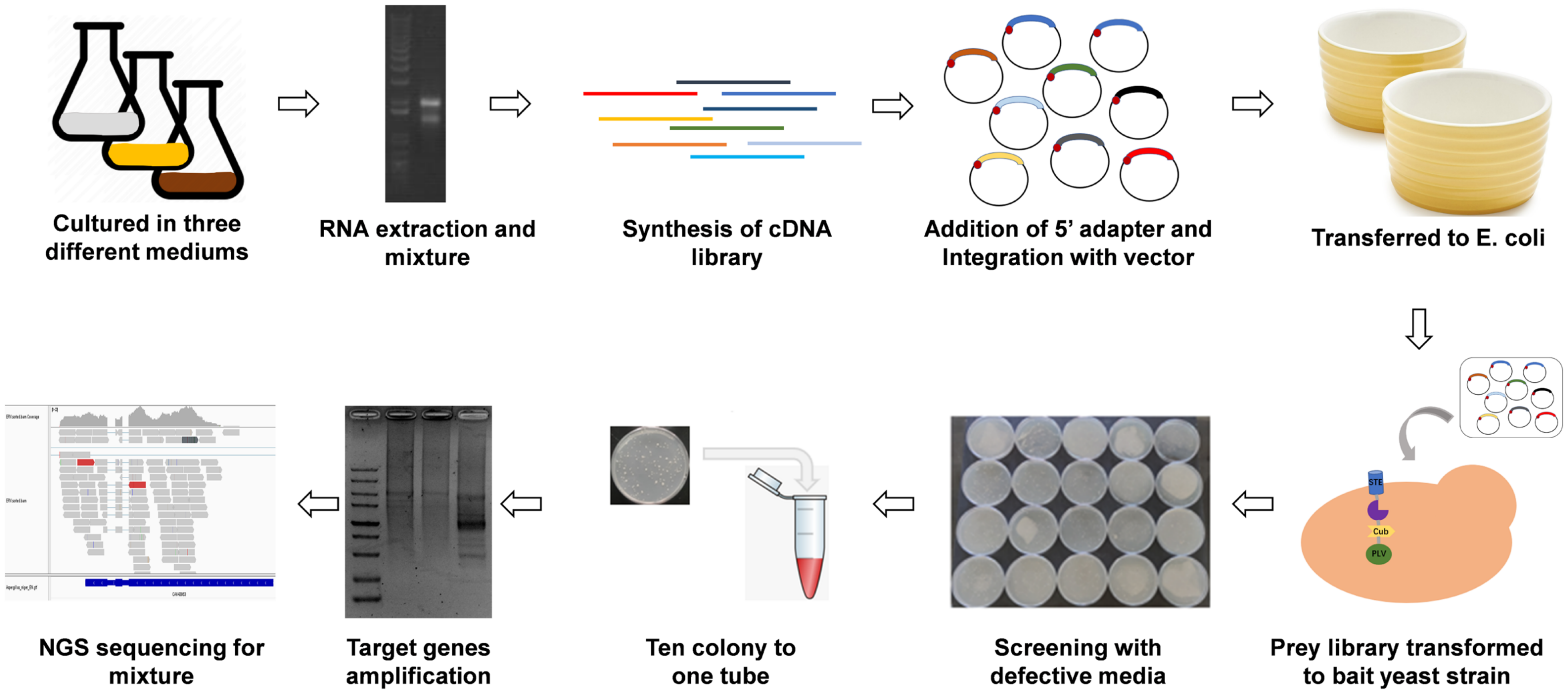
Workflow of the yeast two-hybrid combined with high throughput sequencing. RNA was extracted from *A. niger* cultured in three different media, then cDNA was synthesized and ligated to the prey vector after the addition of the adaptor. The prey library was transformed into the bait strain and then painted in amino acid-deficient media. For high-throughput screening, ten transformants were picked in one tube within 50 μL ddH_2_O, which was used directly as a template for PCR. Then the target genes were amplified. Finally, all the PCR products were mixed for NGS sequencing.

The transport of cargo proteins depends on receptor recognition and vesicle assembly. To fully characterize the proteins associated with *Erv14* transport in *A. niger*, we need to construct a high-throughput screening method. Y2H is a well-established genetic tool for identifying protein–protein interactions, but it has limitations (Rao et al., 2014). To improve the throughput of the assay, we combined Y2H and NGS techniques. The workflow was showed in Figure 3. First, cDNAs from different culture conditions were extracted and used to construct a prey library. We then examined the quality of this prey library. The concentration of the library was found to be 2.3 × 10^6^ CFU/ml by colony counting. The PCR results of randomly selected 96-well colonies showed a 97% positive rate with an average insert size of 1,200 bp. To test the library coverage, the library plasmids were sequenced by NGS. The results showed that the library contained a total of 6,057 protein-coding genes located at various locations within the cells (Supplementary Figure S2). Any gene of interest could be cloned on the bait plasmid. Following the transfer of the bait plasmid into the host and testing for not self-activation, the library plasmids were transferred into the host. Transformants containing both the bait plasmid and the prey plasmid were able to grow on SD-Leu/Trp/His/Ade medium. Each strain from the transformation plate was picked onto the amino acid-deficient medium and then every 10 colonies were mixed into one well for PCR. Finally, the resulting mixed PCR samples were sequenced by NGS.

**Figure 4:**
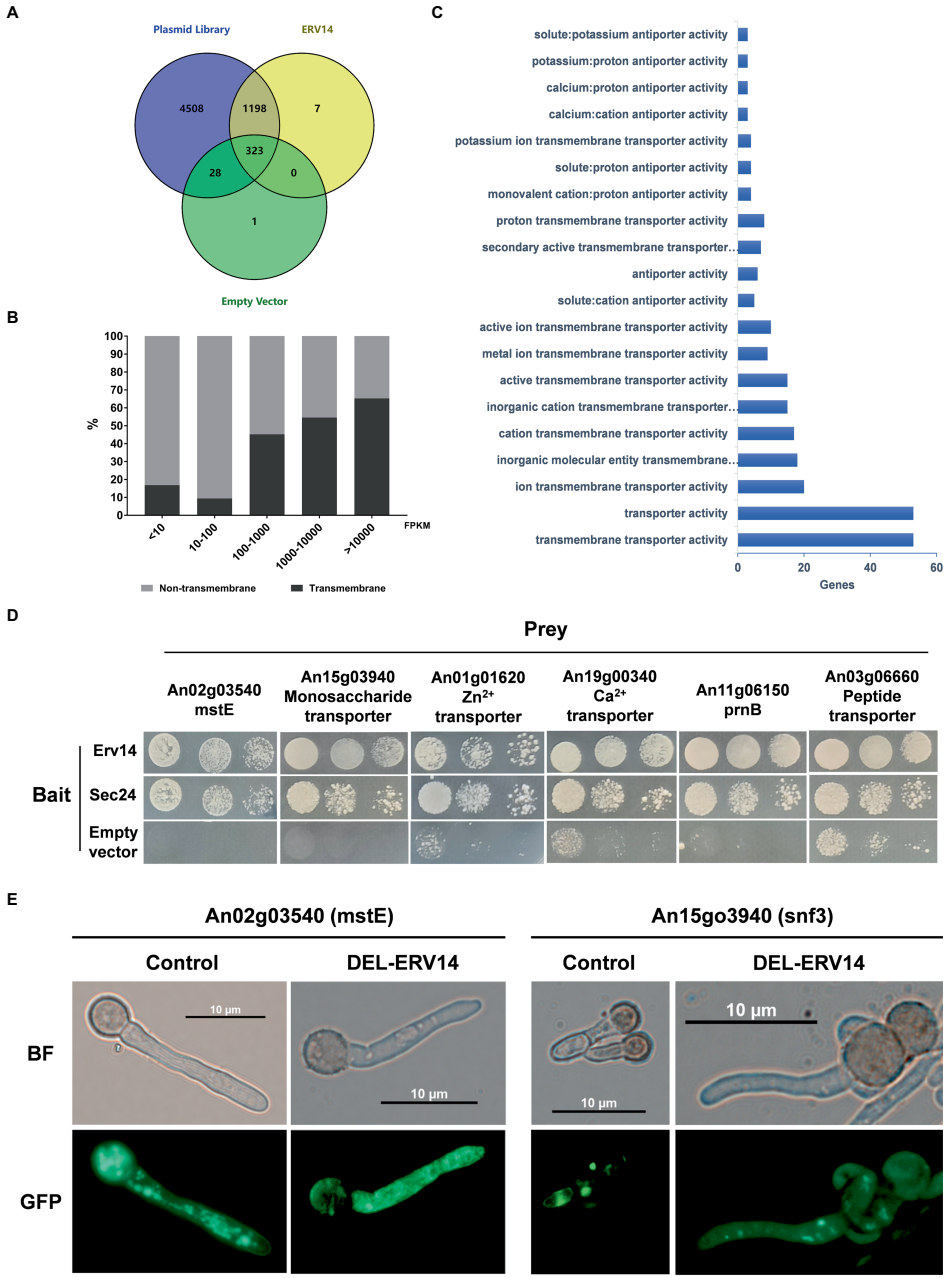
Identification of transporters potentially transported by ERV14 by Y2H high-throughput screening. **(A)** Venn diagrams of the number of overlapping proteins identified in the plasmid library, the empty vector, and the ERV14. **(B)** The proportion of transmembrane and non-transmembrane proteins in the ranges of different FPKM values. **(C)** Functional clustering of “Molecular Function” for transmembrane proteins in the ERV14 interactome. GO distribution of annotated *A. niger* genes was generated using g: Profiler [g: profiler – a web server for functional enrichment analysis and conversions FIGURE 4 (Continued)of gene lists (ut.ee)]. **(D)** Point-to-point Y2H validation of potential interaction between the transporters and ERV14. Six transporters with different functions were selected from the hybridization screen and constructed on prey vectors, which were co-transformed with the ERV14 bait vector. 10 μl of culture solution was spotted on the SD-Leu-Trp-His-Ade solid medium in serial 10-fold dilutions. Sec24, the gene encoding the cargo adaptor, was also constructed on the decoy vector and used as a positive control. The empty bait vector was used as the negative control. **(E)** Localization of monosaccharide transporters in the control strain and the DEL-ERV14 strain.

Following the above approach, we used *Erv14* as bait to screen for proteins interacting with it in the library. As the negative control, we also co-transformed the empty bait vector with the prey library. Unexpectedly, transformants also grew on the screening plate even in absence of any receptor genes. We washed down all these transformants and sent them for sequencing as well, which would be considered as background “Empty” (Supplementary Figure S3). Mapping the sequencing reads to the genome, we identified 1,528 potential Erv14-interacting proteins, 323 of which were also present in the “Empty” (Figure 4A). To eliminate the background, we calculated the FPKM value from the mapped reads and length of each gene. There were many genes with high FPKM values in the background - often those genes that are highly expressed by *A. niger* itself - probably transferred into the same host along with other genes. Then we used the FPKM (Fragments Per Kilobase of exon model per Million mapped fragments) values of each gene in the background as the baseline and calculated the fold change and value of *p*. Only those genes of logFC_Erv14/EMPTY_ greater than 1.5 and *p*-value less than 0.05 were accepted. We finally obtained 1,276 proteins that interacted with *Erv14* after filtering (Supplementary Table S3). Of these proteins, 239 were predicted to have at least one transmembrane region, while the remaining 1,037 proteins did not. Comparing the proportion of transmembrane and non-transmembrane proteins in each FPKM value range, we found that transmembrane proteins had higher FPKM values (Figure 4B). It is generally believed that the higher the FPKM value of which gene is, the higher his confidence level is. Therefore, we thought that *Erv14* is more likely to interact with these transmembrane proteins. To further resolve the function of these proteins, the GO distribution of annotated genes was generated using g: Profiler (Supplementary Table S4). The molecular function of the genes that interacted with *Erv14* was enriched in various transporter activities (Figure 4C). To verify this result, we selected different types of transporters to construct prey vectors for point-to-point hybridization, including monosaccharide transporter, ion transmembrane transporter, and amino acid or oligopeptide transporter. The COPII complex subunit *Sec24* (*An08g10650*) was reported to act as a cargo adapter to interact directly with transmembrane proteins, so it has also been constructed on bait vectors to detect interactions with transporters. We found that both *Erv14* and *Sec24* can interact with all six transporters (Figure 4D). To further verify that the transport of these transporters is associated with Erv14 in *A. niger*, we observed the subcellular localization of two polysaccharide transporters. The mstE and snf3 fused with GFP were overexpressed in the control and DEL-ERV14 strains, respectively. As shown in Figure 4E, although mstE and snf3 were diffusely distributed throughout the mycelium in both strains, they were significantly aggregated at the edge of the mycelium in the control strain, but not in the DEL-ERV14 strain. In a word, we applied a high-throughput Y2H assay to screen 1276 proteins that may interact with *Erv14*, and demonstrated high confidence in the interaction between *Erv14* and multiple transporters by point-to-point Y2H validation and fluorescence localization.

### Membrane proteome analysis of the deletion of *Erv14* in *Aspergillus niger*

**Figure 5:**
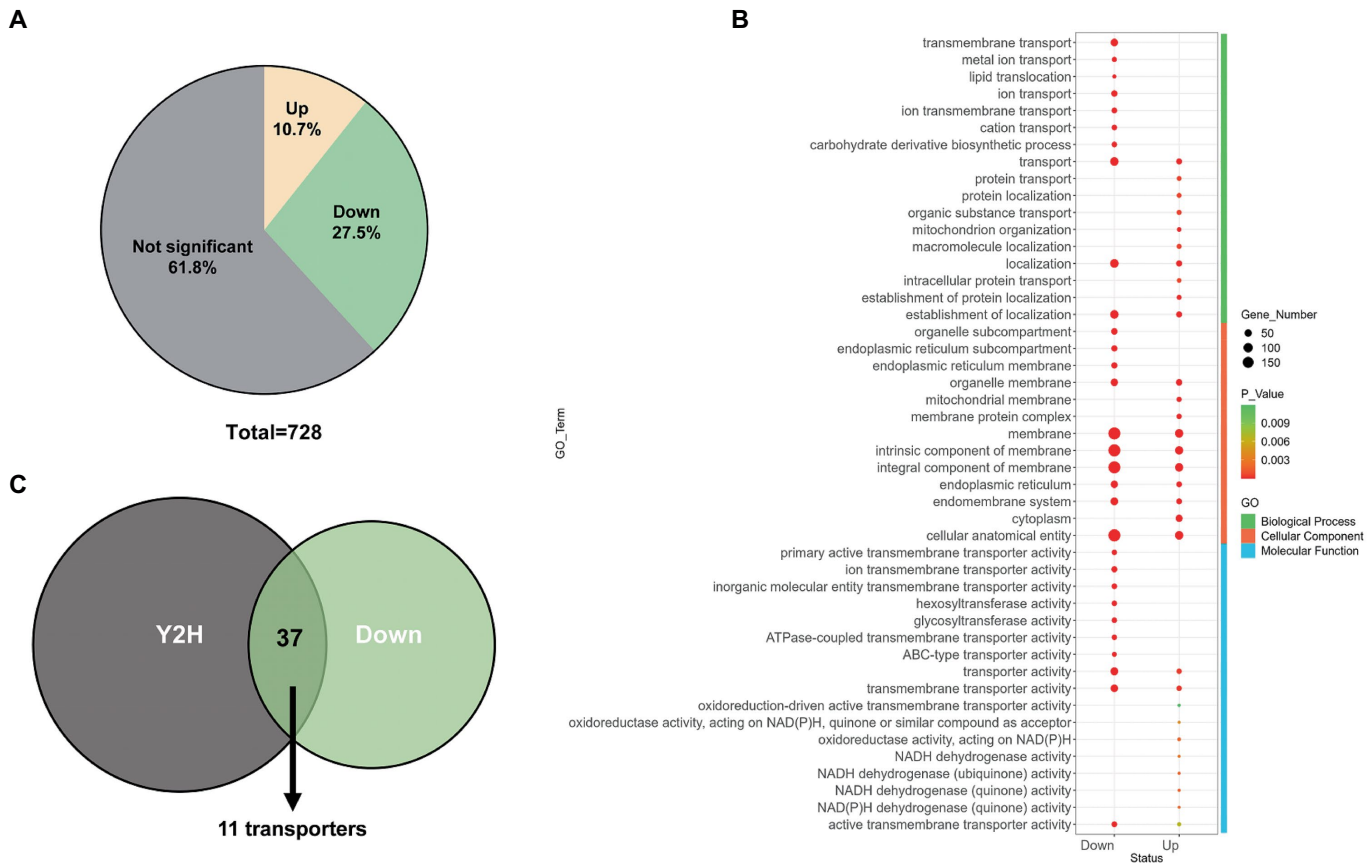
Membrane proteomics analysis of the DEL-Erv14 strain. **(A)** Venn diagram of the proportions of significantly up- and down-regulated membrane proteins in the DEL-Erv14 strain compared to the control strain. The significant difference was defined as log_2_FC greater than 1 or less than −1, and the value of p less than 0.05. **(B)** Functional clustering for up- and down-regulated membrane proteins. **(C)** Venn diagrams of the number of overlapping transmembrane proteins between the ERV14 interactome and the down-regulated proteins identified in the DEL-Erv14 membrane proteomics.

**Figure 6:**
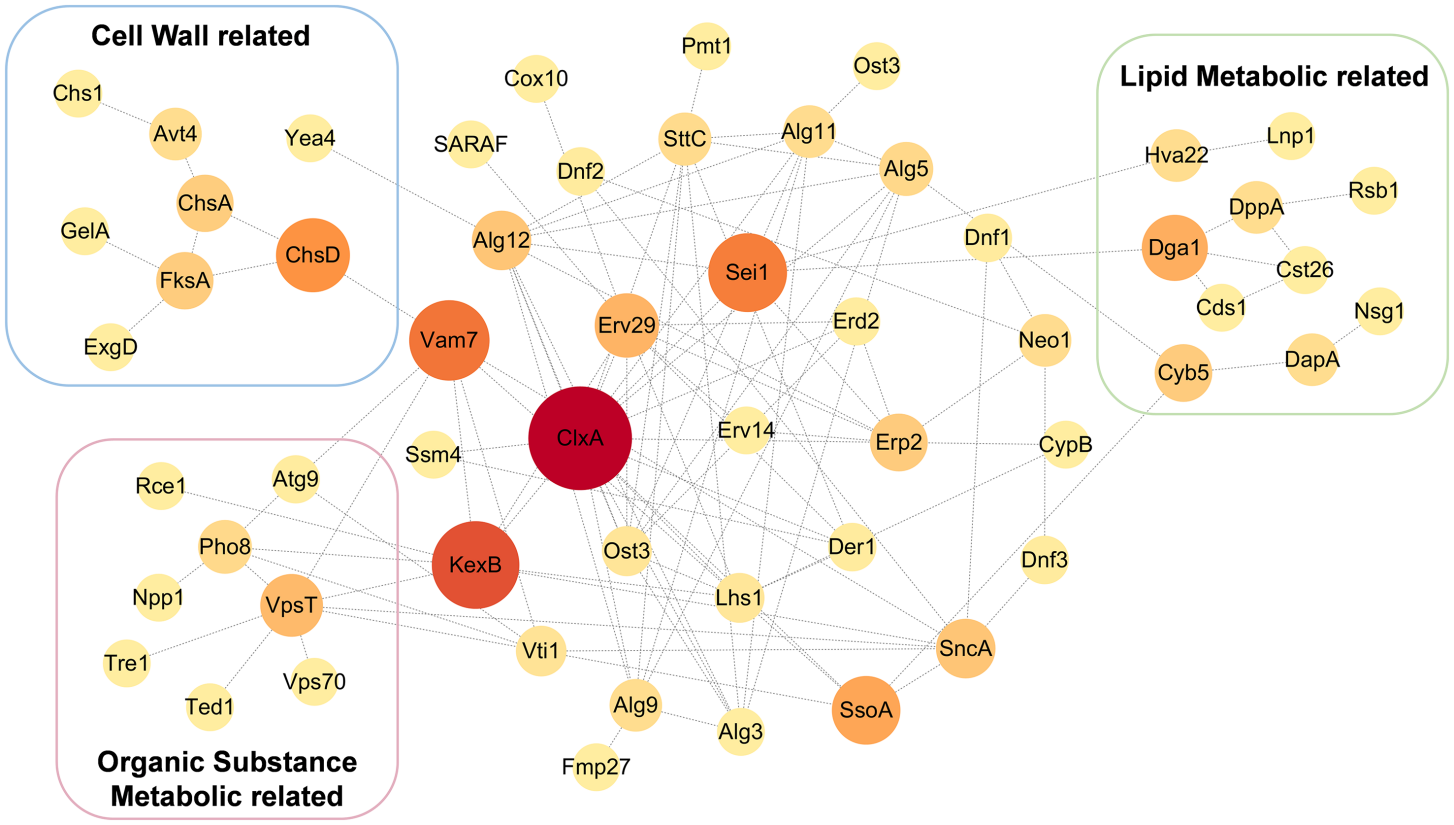
Predicted protein–protein interaction network of down-regulated membrane proteins in the DEL-Erv14 strain. The prediction was performed by STRING [STRING: functional protein association networks (string-db.org)] and exported into Cytoscape for further annotation. The size and color of the bubbles were determined by betweenness centrality (BC).

Since *Erv14* could interact with many transporters *in vitro*, membrane proteins of the DEL-Erv14 strain were analyzed by mass spectrometry to reveal whether the membrane proteins were reduced with the absence of Erv14 in *A. niger*. Three independent cultures of the deletion and the control strains were extracted (Supplementary Figure S4). And the quantitative membrane proteome study was performed by Sequential Window Acquisition of all Theoretical fragment ions(SWATH)technique. A total of 728 membrane proteins were determined in all strains. Among them, most proteins were not significantly different, but the abundance of 78 proteins was significantly up-regulated and 200 proteins were significantly down-regulated (Figure 5A). The up-regulated proteins were mainly enriched in endomembrane systems and were associated with NADH dehydrogenation. And among the down-regulated proteins, the majority were located in the cell membrane and endoplasmic reticulum, exercising transporter function, which was consistent with the functions of the proteins that we identified in the Y2H screening (Figure 5B; Supplementary Figure S5). In addition, *Erv29*, another receptor of the Erv family, and *Erp2*, a receptor of the p24 family, were found to have decreased protein amounts. The expression levels of vesicle transport-associated SNARE proteins *SsoA* and *SncA* were also impaired (Supplementary Table S5). Comparing the proteins down-regulated in the membrane proteome to those screened by Y2H, we found 37 proteins identified by both methods, of which nearly one-third were transporters (Figure 5C). These transporters belonged to different families. Among them, the transporters from the Major Facilitator family (MFS) were mainly responsible for the transport of monosaccharides, while different types of ATPase-coupled transporters were associated with the transport of metal ions. Moreover, there were also permeases, drug transporter and nucleoside transporter, etc. (Table 1). Furthermore, we tried to draw the associated network between *Erv14* and the down-regulated proteins. 57 of 200 proteins were predicted to be capable of forming protein–protein interaction networks by STRING. As shown in Figure 6, *Erv14* was closely related to other proteins located on the ER such as other receptors, and molecular chaperones, which then dispersed to vesicular transport-related proteins and eventually associated with functional proteins. Among these proteins, *chsA (An09g02290)* and *chsD (An09g04890)* were known as chitin synthases, and *fksA (An06g01550)* and *gelA (An10g00400)* encoded glycoside synthases, which were associated with fungal-type cell wall biogenesis process. Additionally, several putative peptidases (*An14g03420, An02g06330, An18g03980*) and alkaline phosphatase (*An07g07520*) were thought to be associated with the organic substrate metabolic process. Another cluster of processes related to lipid metabolism includes acyltransferase (*An13g00040*), diacylglycerol pyrophosphate phosphatase (*dppA, An02g01180*), Cytochrome b5 (*An11g04370*), phosphatidate cytidylyltransferase (*An07g09570*). In conclusion, we identified many proteins with reduced abundance due to *Erv14* deletion by quantitative proteomics, which was associated with vesicle transport, cell wall synthesis, and lipid and organic substrate metabolism.

**Table 1:** Transporters identified in the down-regulated proteins of proteome and the Y2H screening.

Family	Protein accession	Gene ID	Description
MFS family	CAK47634	An02g03540	Fructose transmembrane transporter
	CAK97262	An15g03940	Monosaccharide transporter
	CAK37108	An01g07860	Sulfate transmembrane transporter
ABC family	CAK43073	An17g01770	ATPase-coupled transmembrane transporter
F-ATPase family	CAK37765	An02g08020	H^+^-transporting ATPase lipid-binding protein
P-ATPase family	CAK41966	An14g02290	ATPase-coupled cation transmembrane transporter
AAAP family	CAK38039	An03g00640	–
Ctr2 family	CAK41223	An12g07730	Copper ion transmembrane transporter
DMT family	CAK44946	An06g00300	UDP-galactofuranose transporter
ENT family	CAK44707	An04g07070	Nicotinamide riboside transmembrane transporter
H^+^-channel family	CAK41894	An14g00950	Ferric-chelate reductase

## Discussion

Vesicle trafficking is the primary mode of transport of nascent proteins from the ER to the Golgi apparatus. The recognition and capture of cargo by the receptors determine the early secretory pathway in an efficient and orderly manner. Although the role of the receptors has been well characterized in mammals and yeast, the function of these proteins in *A. niger* was scarcely known.

Phylogenetic analysis shows that receptors are widespread across eukaryotes but vary in number among species. The most obvious difference is the number of receptors in the p24 family. Only four receptors from their respective subfamilies are present in filamentous fungi, whereas 10 receptors are usually present in other species and even in *S. cerevisiae*. Fewer receptors mean that each receptor is likely to carry more cargo for transport. Different from the p24 receptors reported in humans that transport GPI proteins (Lopez et al., 2019), they are also associated with the transport of secreted proteins in fungi. It has been reported that the knockdown of the p24 protein affects the transport of cellulase in *N. crassa* and *P. oxalicum* (Wang et al., 2015; Starr et al., 2018). Our data demonstrate that deletion of all receptors of the p24 family affects mycelial growth and glycosylase secretion in *A. niger*, with deletion of *Erp1* having the most pronounced effect. Members of the ERGIC53 family were first identified in humans as a class of receptors containing lectin domains (Appenzeller et al., 1999; Velloso et al., 2002). In fungi, not only the *Vip36* receptor, which is homologous to that of higher organisms but also the unique *Emp47* receptor are present. And they seem to have opposite functions. In our study, deletion of *Emp47* impairs secretion of glucoamylase, while deletion of *Vip36* improves. Similar results have been found in other *Aspergillus* species. In *A. oryzae*, overexpression of AoVip36 does not affect the secretion of endogenous amylase but will promote the secretion of heterologous fusion proteins. However, overexpression of AoEmp47 inhibits the secretion of endogenous amylase or heterologous fusion protein (Hoang et al., 2015). It has been reported that *Emp47* in *A. fumigatus* is responsible for transporting proteins from the ER to the Golgi apparatus, while *Vip36* acts in protein retrieval from Golgi to ER (Gao et al., 2022). *Erv29* can transport a variety of secreted proteins. In yeast, *Erv29* transports gpαf and CPY by recognizing a hydrophobic signal (Isoleucine- Leucine- Valine, I-L-V) in their pro-region (Otte and Barlowe, 2004). Similar results have been reported in *N. crassa*, NcErv29 is required for the trafficking of the CPY homolog (Starr et al., 2018). MoErv29 promotes the secretion of apoplastic effectors and contributes to the virulence of *M. oryzae* (Qian et al., 2022). In this study, we find that deleting ERV29 reduces the number of spores in the PDA medium and impairs glucoamylase secretion.

Cornichon, a homolog of *Erv14* in higher eukaryotes, is first found in Drosophila to be associated with the orientation of the cytoskeleton during early oocyte development (Roth et al., 1995). In yeast, the deletion of *Erv14* affects the transport of*Axl2*, which causes a defect in polarized growth (Powers and Barlowe, 1998). In our study, the deletion of AniErv14 has the greatest effect on the morphology of the strain. On solid media, colonies become aggregated rather than dispersed. In the liquid medium, the spore-emerging mycelium also does not extend polarly as in the control strain. Deletion of FgErv14 in *F. graminearum* also results in a similar phenotype (Sun et al., 2022). Moreover, we identify some proteins’ role in the establishment of cell polarity in the interactome and proteome, although without further validation. All experimental evidence suggests that *Erv14* does affect the polar growth of mycelium in *A. niger*. Recently, *Erv14* has been reported to be involved in the delivery of cation/proton antiporters and K + -specific transporters (Rosas-Santiago et al., 2016; Zimmermannova et al., 2019). From our results, the deletion of *Erv14* severely impairs the tolerance of the strain to high salt concentrations. And The direct interaction between Erv14 and Zn^2+^ transporter (*An01g01620*) or Ca^2+^ transporter (*An19g00340*) is confirmed by Y2H.

In yeast, Schuldiner et al. develop a systematic approach to pair receptors with cargo proteins (Herzig et al., 2012), and they have identified that deletion of *Erv14* affects the transport of multiple membrane proteins by fluorescence observation. We also want to establish a high-throughput method to systematically identify Erv14-related proteins in *A. niger.* Over the decades, methods for detecting protein–protein interactions have emerged, including affinity chromatography, co-immunoprecipitation, pull-down assays, and yeast two-hybrid (Berggard et al., 2007). Among them, the Y2H assay is widely used because of its convenience and availability, but it also suffers from time-consuming and costly. Combined with next-generation sequencing (NGS), Y2H technology has been continuously improved for large-scale screening (Weimann et al., 2013; Yang et al., 2018; Alcântara et al., 2019). However, the application of Y2H in the investigation of ER receptors has not been reported. A general shortcoming of high throughput screening is the high rate of false positives. These false positives may be caused by multiple prey plasmids entering the same yeast colony, or self-activation. Such as in this study, there are still many transformants growing even if we co-transform the empty bait plasmid with the prey library, which interferes with the accuracy of the experiment. Besides filtering by setting thresholds at the data processing stage as we did, false positives have also been eliminated by continuously passing cultures on selective plates (Vidalain et al., 2004).

In conclusion, we investigated all receptors from the three families and detected their role in mycelial morphology, spore germination, and protein secretion in *A. niger*. Among them, the deletion of AniErv14 severely inhibited mycelial growth and secretion of extracellular proteins such as glucoamylase. Then we established a simple high-throughput Y2H screening method and successfully applied it to detect the potential interacting protein profiles for *Erv14*. By point-to-point re-hybridization, *Erv14* was identified to interact with multiple transmembrane transporters. Quantitative membrane proteomic analysis reveals that proteins implicated by the *Erv14* deletion are mainly related to cell wall synthesis, lipid and organic substrate metabolism.

## Data availability statement

The datasets presented in this study can be found in online repositories. The names of the repository/repositories and accession number(s) can be found in the article/Supplementary material.

## Author contributions

LP: conceptualization and methodology. JZ and LY: validation and data curation. JZ and XZ: investigation. LP and BW: resources, supervision, and funding acquisition. JZ: writing – original draft preparation. LP: writing – review and editing. All authors contributed to the article and approved the submitted version.

## Funding

This work was supported by the National Natural Science Foundation of China (grant number 32270046), the National Key Research and Development Program of China (project no. 2021YFC2100200), the R&D projects in key areas of Guangdong Province (grant number 2022B1111050002), TSBICIP-KJGG-006-21 the Science and Technology Planning Project of Tianjin City (grant no. TSBICIP-KJGG-006-21).

## Conflict of interest

The authors declare that the research was conducted in the absence of any commercial or financial relationships that could be construed as a potential conflict of interest.

## Publisher’s note

All claims expressed in this article are solely those of the authors and do not necessarily represent those of their affiliated organizations, or those of the publisher, the editors and the reviewers. Any product that may be evaluated in this article, or claim that may be made by its manufacturer, is not guaranteed or endorsed by the publisher.

## Footnotes

^1^rgenome.net.
