# Supplementary material for: ERV14 receptor impacts mycelial growth via its interactions with cell wall synthase and transporters in Aspergillus niger
Source: Front Microbiol. 2023 Apr 11;14:1128462. doi: 10.3389/fmicb.2023.1128462 (PMC10126429; doi:10.3389/fmicb.2023.1128462)
Supplement: Supplementary file 1 [file Data_Sheet_1.docx]

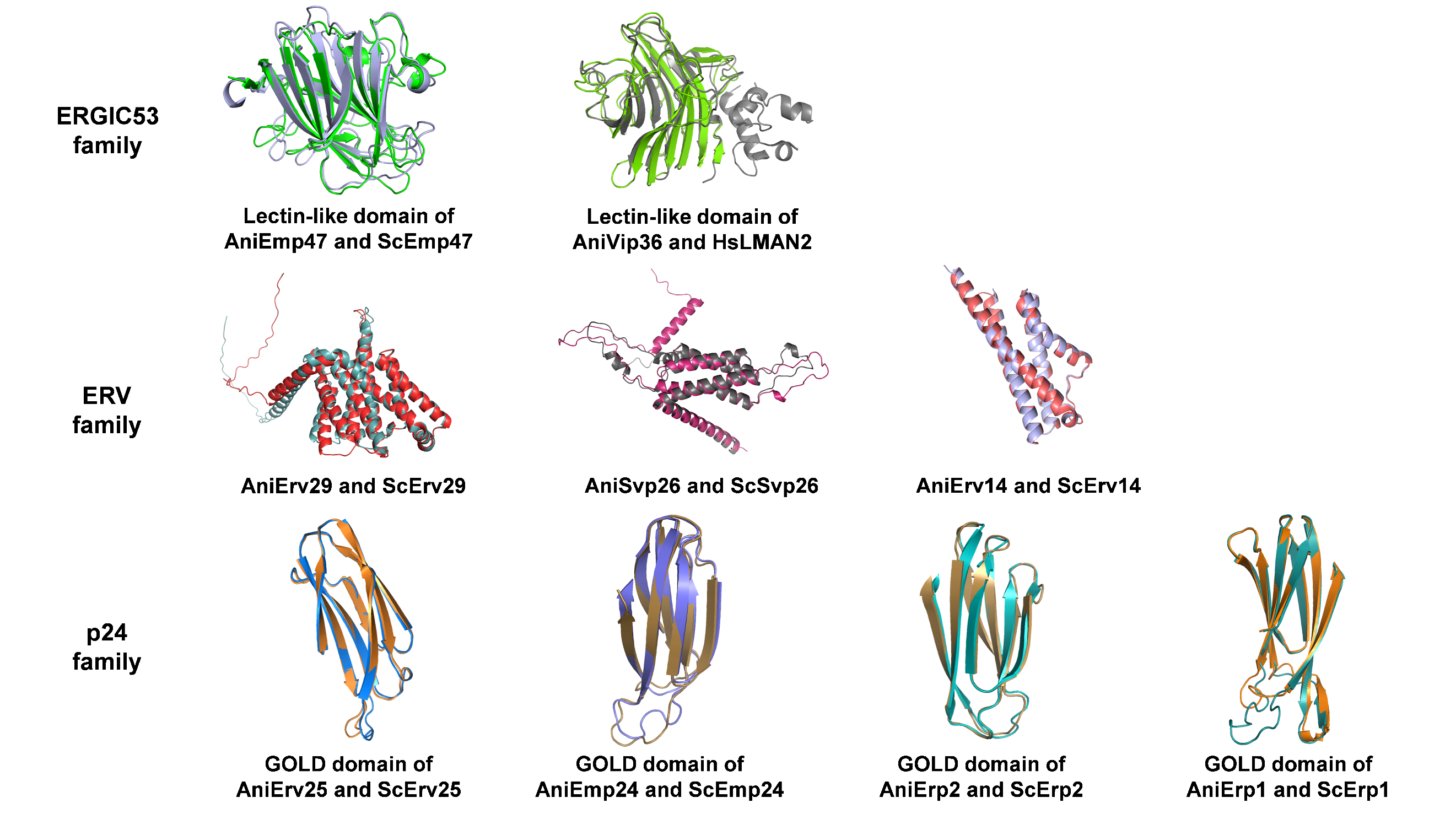


Supplementary Figure S1. Matching the three-dimensional structural model of the conserved domain of each receptor in *A. niger* with that in *S. cerevisiae*. All protein models were predicted by AlphaFold and were matched by PyMol. The Lectin-like domains of AniEmp47 and AniVip36 were labeled with green lines, while those of yeast and human were labeled with gray lines. The three receptors of the ERV family of A. niger were labeled with red lines, while the yeast ones were labeled with cyan lines. The GOLD domains of *A. niger* and yeast p24 family receptors were labeled with blue and yellow lines, respectively.


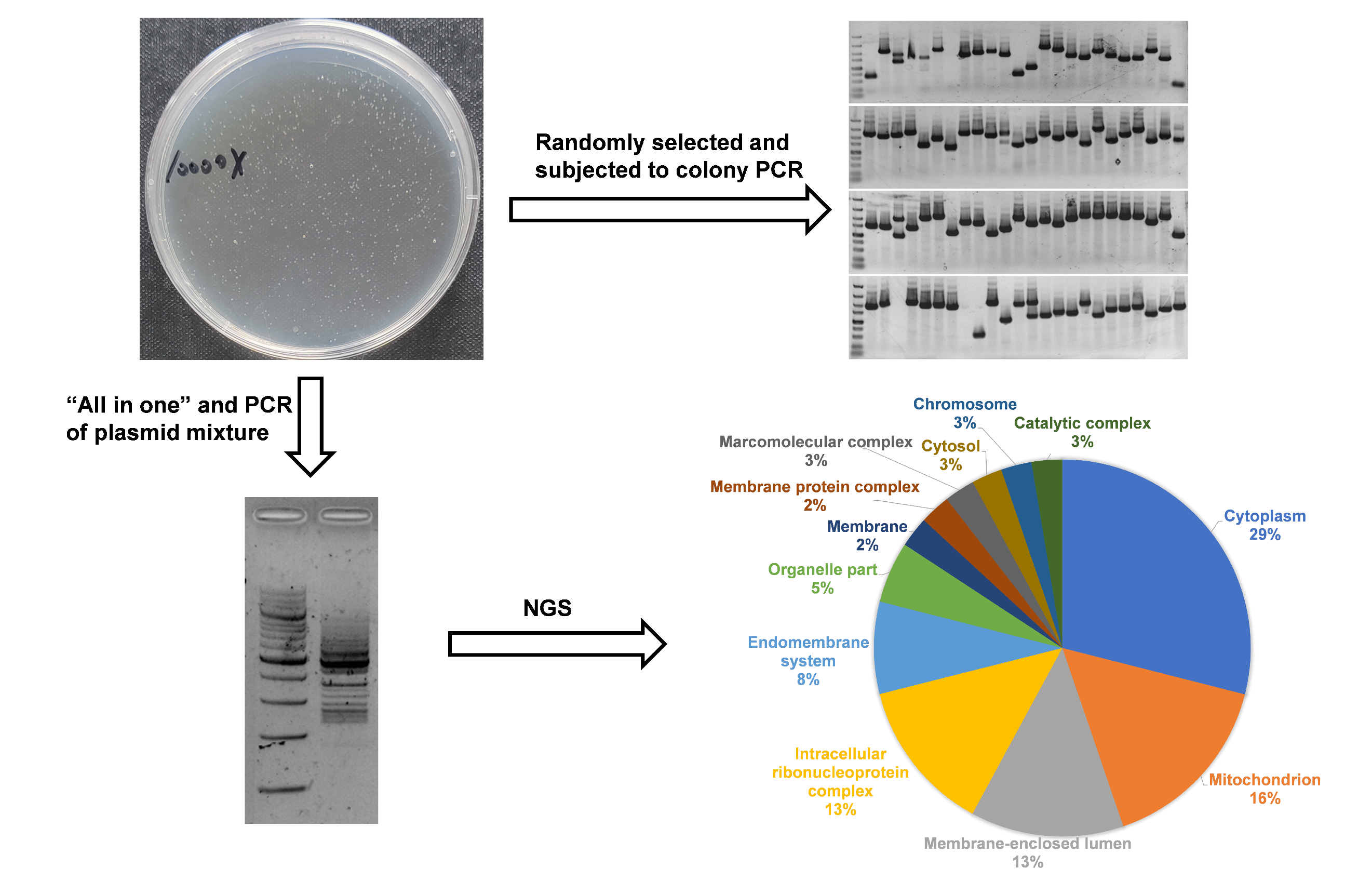


Supplementary Figure S2. Quality testing of plasmid library construction. Library strains were diluted 10,000 times and coated on LB plates containing ampicillin to calculate the library capacity. Ninety-six strains were randomly selected for colony PCR, thus estimating the length of insert fragments and determining the coverage of the library. All transformants were collected for plasmid extraction and then sequenced by NGS. Functional clustering of cellular component for plasmid library. GO distribution of annotated *A.niger* genes was generated using ClueGO(p < 0.05).


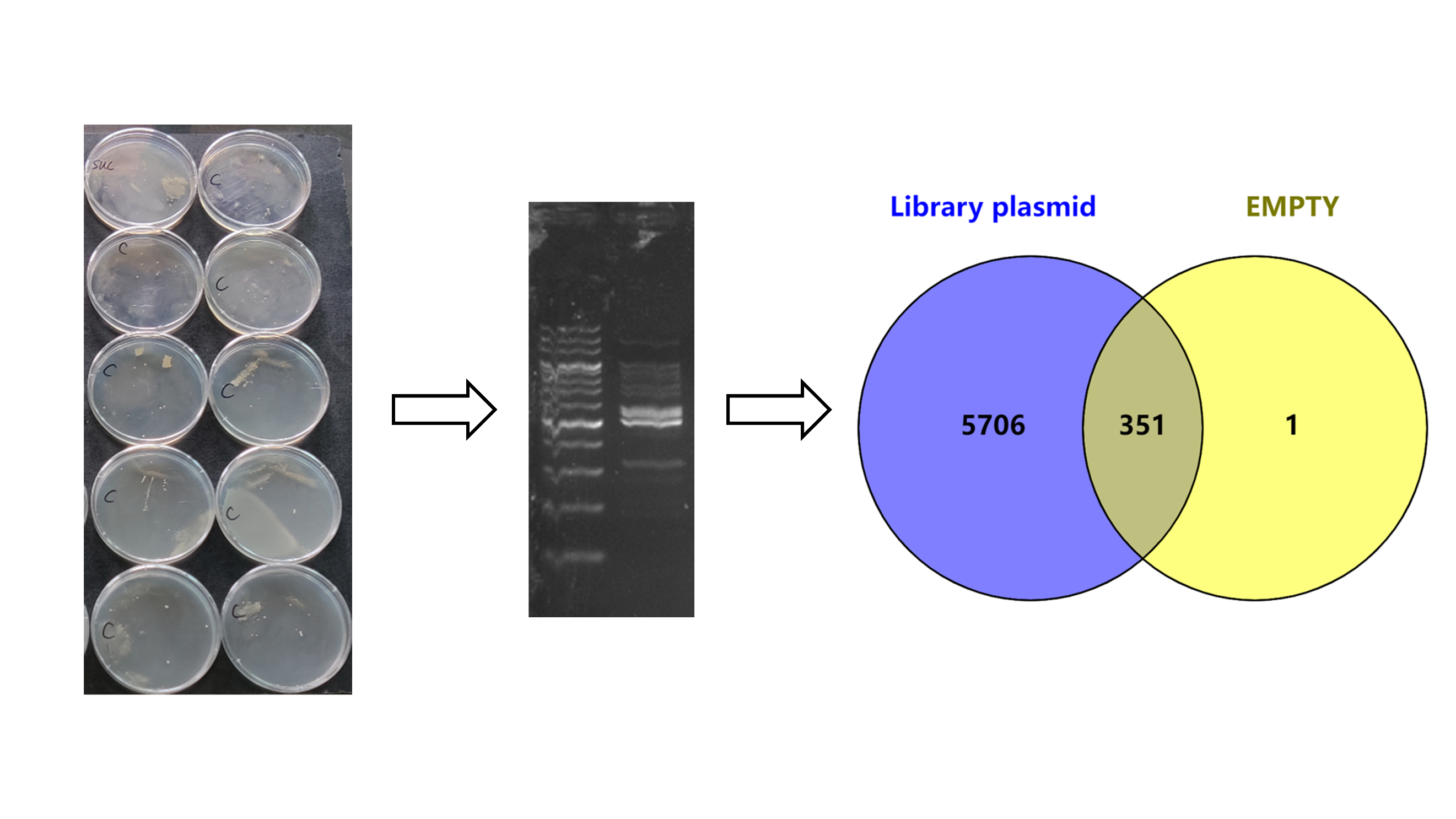


Supplementary Figure S3. Detection of Y2H non-specific backgrounds. The empty bait vector pBT-SUC and the library plasmid were co-transferred into the host strain. All transformants that grew on the defective plates after 4-day culture were picked out and mixed for amplification. Then the mixture was sequenced by NGS.


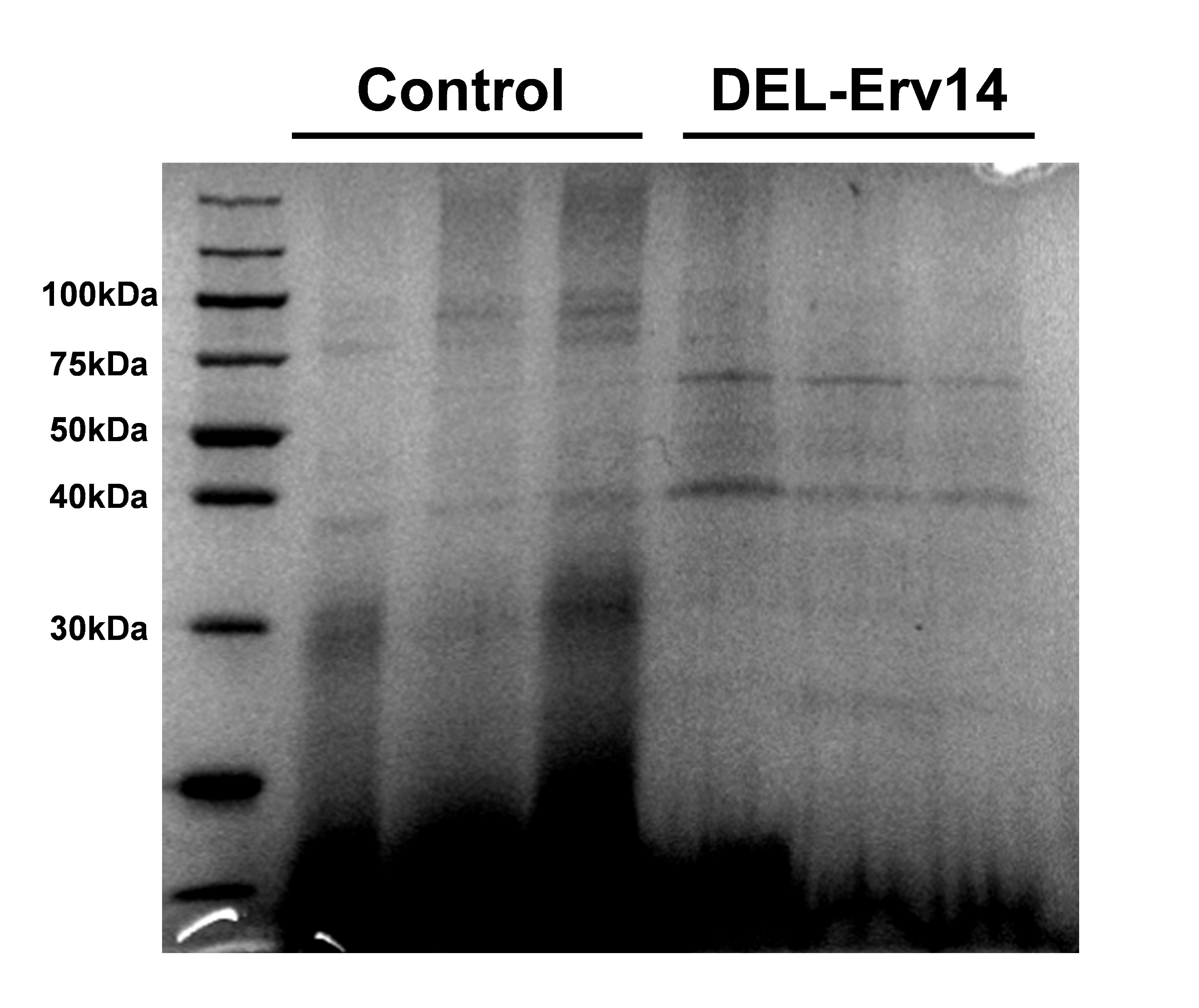


Supplementary Figure S4. SDS-PAGE electrophoresis results of membrane proteins from the control and DEL-Erv14 strains.


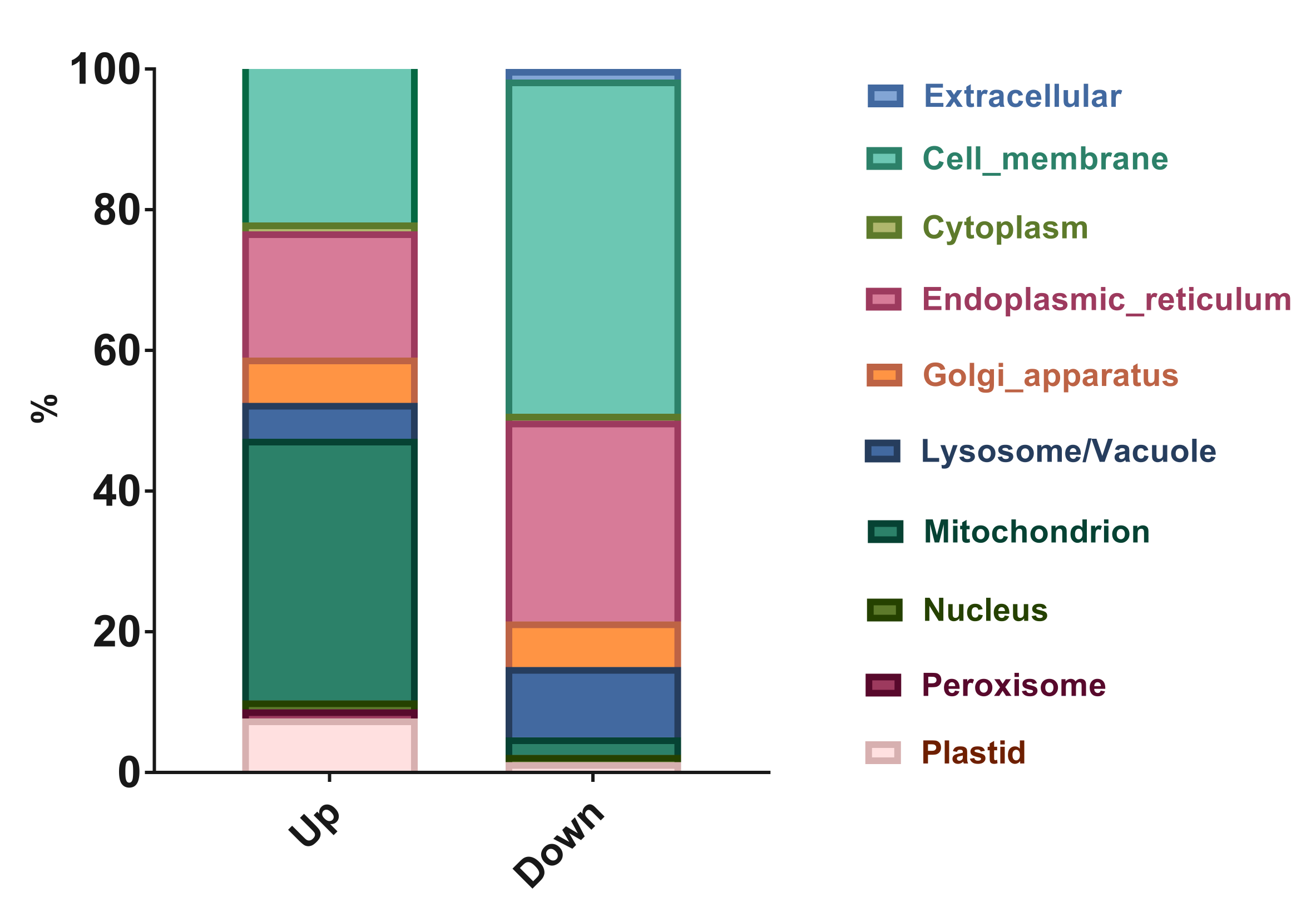


Supplementary Figure S5. The subcellular localization of up- and down-regulated proteins in proteomics predicted by DeepLoc - 2.0([DeepLoc - 2.0 - Services - DTU Health Tech](https://services.healthtech.dtu.dk/service.php?DeepLoc-2.0)).

**Supplementary Table S1 Target sgRNA sequences of each receptor**

| **Receptors** | **Protospacers sequences** | **PAM** |
| --- | --- | --- |
| AniEmp47 | GAAGGAGTCGGGGTTCTCGG | GGG |
| AniVip36 | GGACCTGCAGTACAAGTCCG | AGG |
| AniErv29 | GCAAGCCACCGACAACACTC | AGG |
| AniSvp26 | GCTCCTCCACAAGCTCCGAG | AGG |
| AniErv14 | GGTAGAAGAAGAACATCAGG | AGG |
| AniErv25 | AGAGAGGTCGAACTTCAGGG | CGG |
| AniEmp24 | GTAGTATTGACGGTTACCAA | GGG |
| AniErp2 | GATGGCACCAAGGAAAGACA | GGG |
| AniErp1 | GCAATCCAACACCTACAATG | TGG |

**Supplementary Table S2 Orthologous genes of each family of receptors in mammals, yeast and *A. niger***

| **Receptors families** | | **Orthologous genes** | | |
| --- | --- | --- | --- | --- |
|  |  | **Mammals** | ***S. cerevisiae*** | ***A. niger*** |
| ERGIC-53 | | LMAN1 | Emp47/ Emp46 | An04g08830 |
|  |  | LMAN1L |  |  |
|  |  | LMAN2 |  | An02g04250 |
|  |  | LMAN2L |  |  |
| ERV | | SURF4 | Erv29 | An08g03960 |
|  |  | TEX261 | Svp26 | An18g06740 |
|  |  | CNIH1 | Erv15/Erv14 | An07g09160 |
| P24 | α | TMED11 | Erp1 | An04g01780 |
|  |  | TMED9 | Erp5 |  |
|  |  | TMED4 | Erp6 |  |
|  | β | TMED2 | Emp24 | An08g03590 |
|  | γ | TMED1 | Erp2 | An09g05490 |
|  |  | TMED5 | Erp3 |  |
|  |  | TMED7 | Erp4 |  |
|  |  | TMED3 |  |  |
|  |  | TMED6 |  |  |
|  | σ | TMED10 | Erv25 | An01g08870 |
